# ‘It is not expected for married couples’: a qualitative study on challenges to safer sex communication among polygamous and monogamous partners in southeastern Tanzania

**DOI:** 10.3402/gha.v9.32326

**Published:** 2016-09-14

**Authors:** Sally Mmanyi Mtenga, Eveline Geubbels, Marcel Tanner, Sonja Merten, Constanze Pfeiffer

**Affiliations:** 1 Ifakara Health Institute (IHI), Ifakara, Tanzania; 2 Swiss Tropical and Public Health Institute (Swiss TPH), Basel, Switzerland; 3 Society Gender and Health, University of Basel, Basel, Switzerland; 4 INDEPTH Network, Accra, Ghana

**Keywords:** safer sex, HIV vulnerability, marriage, marital relationship, social norms, communication, polygamous, Tanzania, qualitative study

## Abstract

**Background:**

Behavioral change approaches for human immunodeficiency virus (HIV) prevention in Tanzania encourage married partners to observe safe sex practices (condom use, avoidance of, or safe sex with multiple partners). To implement this advice, partners need to communicate with each other about safer sex, which is often challenging. Although social-structural factors are crucial in understanding sexual behavior, only a few studies focus on understanding safer sex dialogue in a broader social context.

**Design:**

Drawing on the WHO-Commission on the Social Determinants of Health (WHO-CSDH) framework, this study explored key social-structural constructs for studying health in the context of improving safer sex dialogue between polygamous and monogamous partners. Twenty-four in-depth interviews (IDIs) and six focus group discussions (FGDs) with 38 men and women aged 18–60 years were conducted in Ifakara town located in Kilombero district, Tanzania. The study was nested within the community health surveillance project MZIMA (Kiswahili: ‘being healthy’). Partners’ experiences of safer sex dialogue in polygamous and monogamous relations were investigated and the challenges to safer sex dialogue explored.

**Results:**

The study revealed that open safer sex dialogue in marriage is limited and challenged by social norms about marriage (a view that safer sex dialogue imply that partners are ‘not really’ married); marital status (a belief that safer sex dialogue is not practical in polygamous marriages, the elder wife should be exempted from the dialogue since she is at lower risk of engaging in extramarital affairs); relationship quality (marital conflicts, extramarital affairs, trust, and sexual dissatisfaction); and gender power relations (the notion that females’ initiative to discuss condom use and HIV couple counseling and testing may lead to conflict or divorce).

**Conclusions:**

Implementing safer sex practices requires interventions beyond promotion messages. HIV prevention interventions in Tanzania should be carefully adapted to the local context including respective social norms, gender systems, marital context and relationship uncertainties as aspects that facilitate or hinder safer sex dialogue between partners. The WHO-CSDH framework could be strengthened by explicitly integrating relationship quality, marital status, and social norms as additional determinants of health.

## Introduction

Safer sex communication is one of the health protective strategies, effective for the promotion of safer sex practices between partners in stable and marital relations (1–3). Since there is no cure for the human immunodeficiency virus (HIV), promotion of safer sex strategies including safer sex communication remains key in sub-Saharan Africa (SSA) where heterosexual sex is the major route of HIV transmission (4). Safer sex practice is also one of the United Nations’ (UN) 10 priority areas as a cross-cutting strategy in addressing the sexually transmission of HIV (5). However, there is increasing recognition that social-structural context is likely to constrain the practice of safer sex (6–9) including among married partners. Literature highlights that marriages carry taboos that could inhibit safer sex practices between partners (10, 11). These marital taboos are also likely to influence safer sex communication practices in marriage. Recent evidence in Tanzania indicates that the gender norm that a wife is not supposed to ask her husband to use condoms even when he has a disease is common among married partners (12). This norm was significantly associated with HIV status of married men and women in a rural community (12).

Furthermore, alarming HIV vulnerability in marriage as highlighted by studies in SSA including in Tanzania (13–16) is an indication that more data is required on social-structural drivers of HIV vulnerability in marriage. Hence, understanding contextual aspects surrounding safer sex communication between partners is critical for designing appropriate interventions to promote safer sex communication and practices between partners in marriage.

In Tanzania, health promotion messages that encourage married partners to use couple counseling/testing and condoms, as well as to abstain from extramarital affairs have been consistently promoted as HIV prevention interventions for married partners. A widely promoted Swahili slogan is ‘stay on the main road, divergence is not an option – prevent HIV’ (Kiswahili: ‘baki njia kuu mchepuko sio dili-epuka ukimwi’) (17). Implementing the recommended safer sex practices requires partners to communicate with each other about safer sex. This is often challenging despite evidence that communication and negotiation for safer sex play major roles in HIV prevention in SSA (1–3). In India, better safer sex communication led to increased sexual activity, improved relationships, alleviate doubts about a partner's infidelity, and increased forgiveness among married partners (10). In contrast, poor sexual communication has been positively associated with the prevalence of sexually transmitted infections among married men and women (18).

Studies based on psychological and behavior theories have led to insights into variables (i.e. communication skills, attitude, intention, perception, self-efficacy) that influence safer sex communication and practices (18–24). Such studies, however, are criticized for the failure to account for the social-structural context in which the safer sex practices operate (8–10). For instance the main critique of the ‘ABC’ (condom, abstinence, and being faithful) model of HIV prevention is its ‘individualistic’ focus, paying little attention to the context of the safer sex aspects (8, 9, 25).

Different from the psychological and behavioral approaches (18, 19) to safer sex communication and practices, in this paper we take a step further to understand how social-structural aspects including marital experiences challenge safer sex dialogue between married men and women in monogamous and polygamous relations.

Safer sex communication hereby refers to how married partners communicate and negotiate about condom use, couple counseling/testing, and abstaining from extramarital affairs. We confine safer sex to these safer sex aspects based on the ongoing health promotion messages in Tanzania, evidence of their relevance and their low uptake among married partners.

## Theoretical framework

**Fig. 1:**
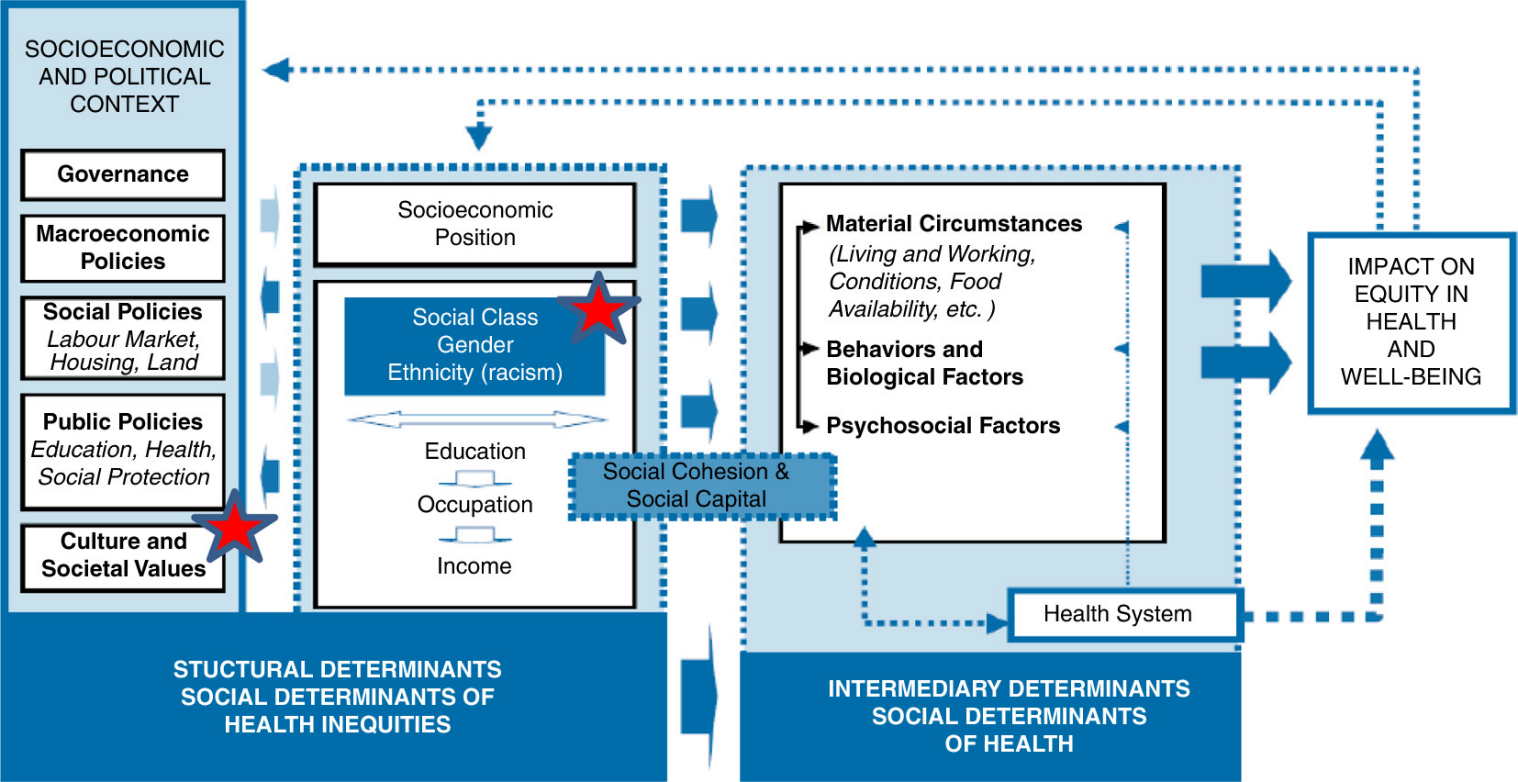
The WHO-Comission on the Social Determinants of Health (WHO-CSDH) framework (2010).

We study safer sex communication in the context of the WHO-Commission on the Social Determinants of Health framework (WHO-CSDH) (26). The primary aim of the framework is to guide understanding and practice in addressing health inequity and diseases (26). Although, the primary aim of the WHO-CSDH framework is not to investigate factors influencing safer sex communication between married partners, the framework includes relevant broader social-structural aspects (Fig. 1) that can be adopted to study health behavior aspects. By focusing on the social, economic, and political context, this study explored cultural and social values, gender aspects, which may affect safer sex dialogue. The red stars in the framework presented below illustrate this focus (Fig. 1).

**Fig. 2:**
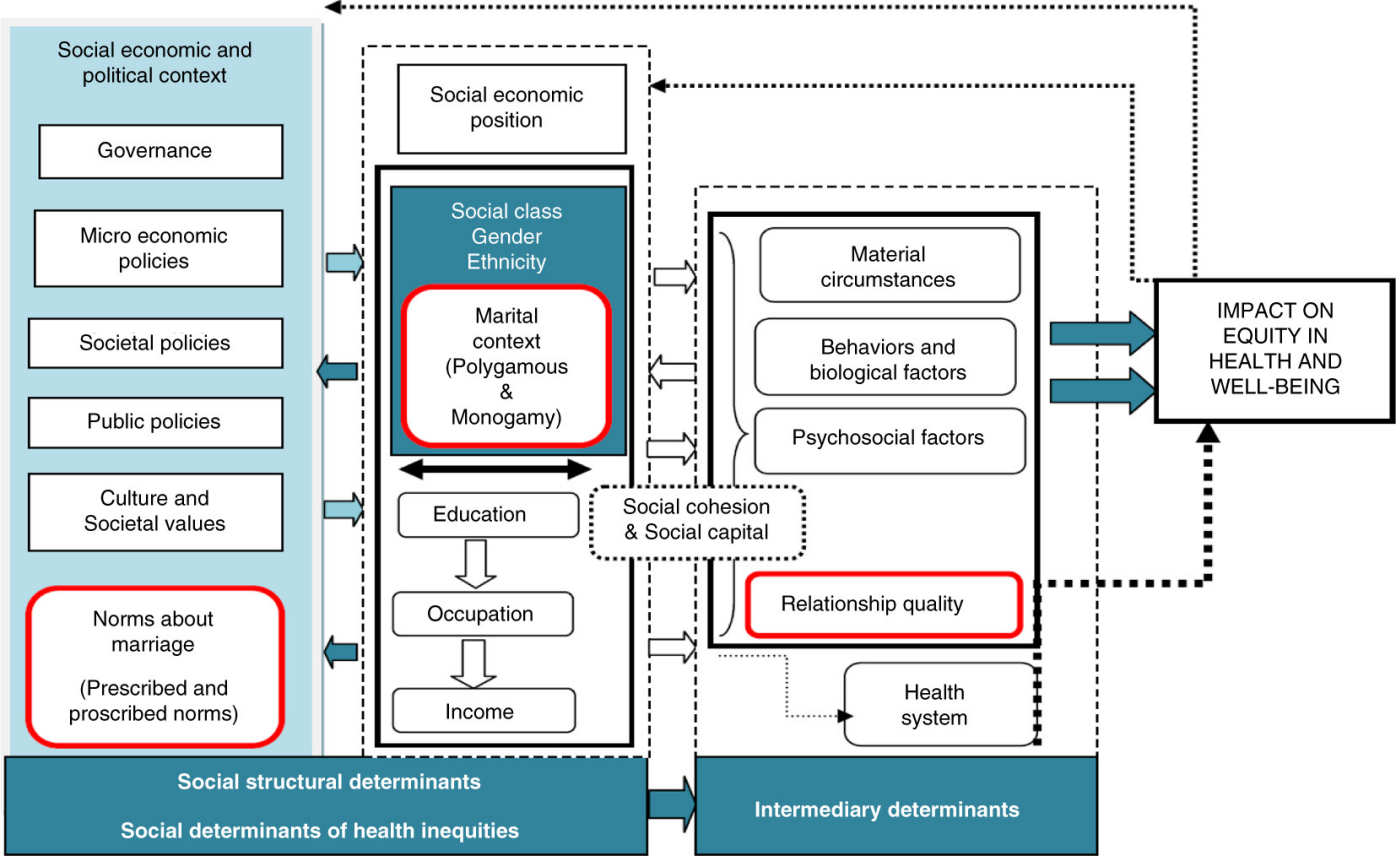
Modified WHO-SDH framework used to learn about the challenges to safer sex dialogue between married spouses. From WHO (26).

Acknowledging the limitations of the framework, based on the literature review and intuition, additional social-structural constructs were explored to improve understanding of safer sex dialogue in marital relations. The red boxes in Fig. 2 below present these additional constructs.

## Definition of key concepts

### Social norms

Jackson (27) defines social norms as rules endorsed in accordance with the social-cultural expectations. Norms are representations of acceptable group conduct (28) motivating people to conform to rules (29). Prescriptive norms are unwritten rules indicating what one should do while proscriptive norms indicate what one should not do (30, 31). Bicchieri (32) states that norms are important social constructs since they are endogenous products of individuals’ interactions and imply punishment or negative social consequences if one fails to comply. Hence, they are likely to shape health practices (33). Both types of norms were explored in this study.

### Relationship quality

Relationship quality is often referred to as how happy or satisfied partners are in their relationship (34, 35). Approaches to relationship quality comprise of ‘interpersonal relationship’ (patterns of interaction between spouses: communication, conflict behaviors, and spending time with one another) and ‘intrapersonal approach’ referring to how partners view happiness or satisfaction in a relationship (34). In most studies ‘trust’ between partners and ‘satisfaction’ in a relationship have been the main measures of relationship quality (36). Poor interpersonal and intrapersonal relationship quality is linked to poor health outcomes for partners (37).

### Marital context

Marital context in this study refers to different types of marriage. Polygamous and monogamous relationships are the common patterns of marriage in SSA including Tanzania. About a quarter of the women in Tanzania live in polygamous marriages (38), which refers to one man having more than one wife; monogamous refers to one man having one wife (39). Observation that monogamous and polygamous partners are likely to engage in sexual risk behavior (16) provides indication that both marital types are not protective against HIV infection.

## Methods

### Design

Using a qualitative approach, we conducted 24 in-depth interviews (IDIs), and six focus group discussions (FGDs) with married men and women residing in Ifakara town, Kilombero district in southeastern Tanzania. The IDIs were useful in uncovering individuals’ experiences regarding safer sex dialogue and capturing sensitive, emotive, and salient aspects in marital life which one may not feel comfortable discussing in the presence of other people (40). FGDs allowed insights into general group norms on marriage, or ‘collective’ views, beliefs, and discourses related to safer sex dialogue in marriage. The use of both tools helped to cross-check information (41).

### Study setting

The study was implemented between May 2015 and July 2015 in two villages of Ifakara town: Viwanja sitini and Mlabani in Kilombero district. It is nested within a larger MZIMA community health surveillance study (42). The detailed description of MZIMA surveillance study and Ifakara town is provided elsewhere (12). While MZIMA offers quantitative data regarding predictors of HIV status among heterosexual married individuals, this qualitative study was set up to build upon a previous study focusing on the social drivers of HIV status among married and cohabiting partners (12), to gain a better understanding of the complexities of safer sex dialogue that cannot be easily captured by survey research.

Ifakara town is the district headquarters of the Kilombero district where most of the administrative and social activities are implemented. The town is heterogeneous, hosting more than nine ethnic groups coming from other parts of the country. The three most common ethnic groups are the Wandamba, the Wapogoro, and the Wambunga (12). Islam (39%) and Christianity (52%) are the predominant religions (12). The town is well served by a hierarchy of health facilities ranging from the district hospital to health centers and dispensaries. The adult HIV prevalence in Ifakara based on the MZIMA community surveillance study is 7% (12), which is higher than the national prevalence 5% (16).

### Participants’ recruitment and data collection

We conducted 24 IDIs with married and cohabiting individuals living in heterosexual monogamous and polygamous relations, and six FGDs with married partners (Tables 1 and 2). The sample size for the IDIs was based on the saturation principles (43) recommending a sample size of 12. Each FGD had 6 to 10 participants to achieve a maximum variation.

**Table 1:** Summary of the IDIs participants in Ifakara town (*N*=24)

Participant's characteristics	Total number of participants
Sex	
Males	11
Females	13
Age	
24–30	08
31–36	05
37–42	08
43–48	01
49–54	00
55–60	01
61–67	00
68–74	01
Education	
Never gone to school	01
Primary education	19
Secondary education	02
Higher level	01
Occupation	
Farmers	13
Farmers/business	02
Petty traders	04
Business	04
Teacher	01
Marital type	
Monogamous	15
Polygamous	09
Marital duration	
1–5	09
6–11	06
12–17	05
18–22	03
30	01
Number of children	
0	01
1–3	13
4–6	07
7–9	02
20	01
Religion	
Muslims	07
Christians	17

**Table 2:** Summary of the FGD participants in Ifakara town (*n*=38)

Participants’ characteristics	Total number of participants
Sex	
Males	18
Females	20
Age	
18–25	12
26–34	11
35–43	15
Education	
Primary education	36
Form four	02
Occupation	
Farmers	25
Petty traders	06
Business	07
Marital type	
Monogamous	25
Polygamous	13
Marital duration	
1–5	25
6–11	09
12–17	04
Number of children	
1–3	27
4–6	11
Religion	
Muslims	15
Christians	23

A stratified purposive sampling was used to recruit the IDIs and FGDs participants by ensuring that the younger (18–30 years age group), older (30–60 years age group), polygamous, and monogamous individuals were captured. We assumed that marital characteristics (polygamous, monogamous) would be important in understanding partners’ marital contexts and experiences affecting a safer sex dialogue. During FGDs, we separated individuals according to gender and age but we combined the polygamous and monogamous marital characteristics in the discussion groups due to fewer polygamous partners. Familiarity with the study setting, prior informal discussions with community members and the Swahili language was useful for the first author (SM) in gaining access to the relevant community and the local leaders.

The community hamlet leaders were useful in the recruitment of the study participants. They helped with understanding of marital types in the study area, that is, polygamous, monogamous, and facilitated access to married couples. Semi-structured and open-ended discussion guides were used during the interviews. Four broader topics were explored: 1) perceptions of marital life, 2) perceptions of safer sex methods, 3) experiences of safer sex dialogue, and 4) community views on safer sex negotiation between partners in monogamous and polygamous relations. The guides were pre-tested and afterwards revised. The study investigator (SM) (a female married Tanzanian adult and a sociologist) with experience in qualitative data collection carried out the study.

A married female research sociology graduate assisted in conducting interviews and discussions with a married male participant. The study venues were selected based on participants’ preferences, that is, under the trees, at private places, at homes, in their neighborhoods, and at school classrooms. Participants were interviewed until no new insights emerged which was observed at the 24th interview. To enable free narration of personal experiences and emotional sentiments, we recruited partners from different households. We did not interview participants based on the background of their sexual risk behaviors (i.e. condom use, having multiple partners) since we intended to capture the broader marital relationship and lifestyle.

All participants were asked for written informed consent after being informed about the purpose of the study. Confidentiality was assured and participants were informed about their right to withdraw from the study at any time. Iterative and recursive processes in data collection were employed to explore emerging concepts or themes from one interview to the other (iterative) or going back to check for the issues raised in the previous interviews (recursive) (44). Interviews and discussions were conducted in Kiswahili, a debriefing session was conducted to review emerging issues and prepare for the subsequent interviews. Participants were given a small grocery package as compensation for transport, that is, hiring of bicycles to the interview venue. Data were audio recorded and transcribed verbatim.

### Data analysis

Building on the principle of grounded theory (45), the verbatim-transcribed data were analyzed. Emerged patterns were identified and coded. Coding began as open coding assigned next to the study themes or ideas found in segments of the transcript. Inductive and deductive codes enhanced the analytical meaning of the emerged themes (46). Themes were compared between men and women (young and old) and monogamous and polygamous relations.

Tentative themes developed from the analytical process were compared with others to check for validity. Themes, categories and subcategories were guided by the social determinants of health framework (26). A summary of the aspects emerging from the analytical process is provided (Table 3). N-VIVO program version 12 for qualitative data analysis was used to support systematic data coding. All data were analyzed in Kiswahili, and relevant quotes were translated into English.

**Table 3:** Major themes and subthemes that emerged in the study

Major themes	Subthemes
Relationship quality	• Marital conflict
	• Marital trust
	• Extramarital affairs
	• Sexual dissatisfaction
Social norms	• Norms of marital relations
	• Norms of marital status (Polygamous relation)
Gender power relation	• Threats of conflict and divorce

### Trustworthiness

This qualitative study considered credibility, dependability, and transferability (47). After the data analysis, the member checking method was used for validation of data. Participants were asked to check for the accuracy of the generated themes to allow clarity on the interpretation and practicality of various key themes and subthemes. Participants provided feedback, which helped to improve the accuracy of themes.

### Ethical consideration

Ethical clearance was acquired from the Ifakara Health Institute Review Board (approval number IHI/IRB/AM/01-2014), the National Medical Research Coordinating Committee (approval number NIMR/HQ/R.81Vol.IX/1320) and the Ethikkommission Nordwest- und Zentralschweiz in Basel, Switzerland (EKNZ:UBE-15/36).

## Results

### Participants’ characteristics

Table 1 shows the characteristics of married and cohabiting individuals enrolled in the IDI interviews; Table 2 shows the characteristics of the participants of the FGDs. Twenty-four participants (13 females and 11 males) participated in the IDIs. Thirty-eight individuals tool part in the FGDs (18 males, 21 females). Participants were aged between 18 and 68 years. All participants were married, 44 lived in monogamous marriages and 20 in polygamous relations. In polygamous marriages, the number of reported co-wives varied from two to four. Participants reported to have had one to seven children, and had been married for 10 years or less (Tables 1 and 2).

### Challenges to safer sex dialogue

Three main themes highlighting the challenges of safer sex dialogue are presented below (Table 3). By focusing on the relationship quality, we start by presenting partners’ views about their marriages as ‘happy marriage’, and how this view is challenged by marital uncertainties. We then elaborate other key themes, which challenge safer sex dialogue between partners. Lastly, we present how participants communicate and perceive safer sex.

We expected to observe strong divergences of opinions between polygamous and monogamous partners. However, the opposite was found. Differences were only noted with regards to norms related to the marital status.

### Relationship quality

#### Happy marriage identity

At the start of the interviews, various participants (men and women) characterized their marriages as being ‘happy’. Later it became clear that a happy marriage does not necessarily preclude one of the partners from having extramarital sexual relationships or conflict. One male participant reported that he is happy with his marriage and that his wife treats him well but in the course of the interview, he disclosed to have had several extramarital partners:

Now days without marriage you will be hanging here and there but if you have your wife you just stay and enjoy, so when I see my wife I feel very happy.

What really makes me practice extramarital affairs is lust, just lust because the lady (extramarital partner) is beautiful, have nice shape and tall […] she was not married and just finished her form four.

[IDI_Male_38 years_Monogamous]

Some women revealed being happy in their marriage but they reported to engage nevertheless in extramarital behavior. It could be a reflection that living in a ‘happy marriage’ is a ‘valued identity’ among marital partners (both men and women). However, this identity is affected by the circumstances within marriage (extramarital affair). One female participant expressed her experience:

I love my marriage, I cannot quit, if my husband's behavior improves I will be happy to continue with my marriage.

At first I did not have a man outside my marriage, but I was forced to do that after seeing that my husband does not pay attention to my needs and he also does the same (engage in extramarital affairs). I have been with this man (extramarital partner) for two years now, he is not married […] we use condoms but not always.

[IDI_Female_24 years_Monogamous]

#### Marital conflict

Participants frequently mentioned marital conflict as an aspect that dominates their marital lifestyle. One female participant explained that marital disharmony affects mutual dialogue on safer sex since it becomes impossible for partners to talk. She confessed frequent quarrels with her husband:

P: If there is marital disharmony, you cannot talk about anything. If you always quarrel on things, you cannot agree on things (including safer sex practice). Where there is no love, these discussions are impossible.

I: You have talked about quarrels does this happen in your marriage?

P: Yes, very often, I tell him (husband) do not come back late at night and do not have other partners but he does not listen.

[IDI_Female_40 years_Monogamous]

Similarly, a male participant pointed out that absence of happiness in the marriage makes it difficult for partners to practice safer sex dialogue since they cannot stay together and plan:

I: What do you think are the reasons that couples do not communicate about safer sex?

P: They do not have good relationships because marriage is happiness if you are not happy you will not discuss (about safer sex aspects).

[IDI_Male_38 years_Monogamous]

#### Marital trust

The concept of ‘trust’ was evident in most interviews and discussions. Partners expressed their strong opposing views towards dialogue about couple counseling and testing or condom use since this contradicts trust in marriage:

When people have been married for a long time, they become like relatives and you trust each other and it is difficult to talk about condoms or going for HIV testing since you already trust each other.

[FGD_Males_01]

You know, when you agree to live as husband and wife you need to trust each other and be ready for anything (not having safer sex dialogue).

[FGD_Females_03]

#### Extramarital affairs

Participants (particularly women) frequently cited an extramarital affair as one of the constructs that affects marital happiness and sexual passion in marriage and may hinder mutual dialogue on safer sex.

One woman explained that she would be happy to see her husband abstaining from extramarital affairs since the behavior affects happiness and the quality of sex in her marriage:

P: I would feel happy when my husband does not have outside sexual affairs

I: Can you tell me more, why do you say so?

P: Because if a man has other women outside marriage (extramarital affairs) there will be no happiness in the house […].

I: Why do you think that he (husband) has partners outside marital?

P: My husband usually tells me I am tired when I ask for sex […]

[IDI_Female_40 years_Monogamous]

A male participant admitted that he always quarrels with his wife due to his extramarital behavior:

The main issue that we normally have conflict with is about women (extramarital women), she (wife) always think that I have other women, but sometimes I have (extramarital women) sometimes not.

[IDI_Male_26 years_Monogamous]

#### Sexual dissatisfaction

Participants also talked about how it may be difficult for the partners to implement safer sex communication since some spouses are not sexually satisfied in their marital affairs.

One participant explained how the husband has been denying sex for a long time:

Men are the source of the problem (not having safer sex dialogue); I do not know why the society does not see this. On my side, I have been very much patient with my husband, I stayed for one month, two months without getting sex from him. When he comes home he just sleeps and turns the back on a different side.

[FGD_Females_01]

A man in a polygamous relation highlights a similar concern:

You find that I need sex frequently but she refuses to give me, whenever I need (sex)

she fails to satisfy me.

[IDI_Male_41 years_Polygamous]

### Social norms

#### Norms of marital relations

Some participants were of the view that safer sex dialogue between married partners is not acceptable in the Tanzanian community. This shared belief could be one of the explanations for the low uptake of condom use and couple counseling and testing among married partners.

Here people feel shy to address the reality. To speak the truth, in our normal Tanzanian communities for a husband and a wife to sit together and talk about HIV prevention or condom use or HIV testing is not common and neither expected, unless you suspect each other. Even when other people in the community hear that Mr. so and so and the wife talk about these issues they may think that you are not in real marital relations.

[FGD_Males_02]

Some study participants stated that if married partners are seen by community members talking about HIV prevention, people may start doubting whether they are real in a marital relation. These sorts of discussions are meant for single and young people:

Sometimes people (in the community) may question, why do these married people talk about HIV prevention? I think that, those discussions are for the single and young people. Like me if my husband tells me about condoms or HIV prevention, I may think that he does not respect me as a wife or suspect about issues.

[FGD_Females_03]

Another participant had the opinion that it is against the law to advise a spouse to use a condom with an extramarital partner and may look like approving the extramarital behavior:

It is not legal to tell your wife that use condom when having extramarital affairs, it is like allowing her to go outside. I can never propose condom use with my wives.

[IDI_Male 56 years_ polygamous]

#### Norms of marital status (polygamous marriage)

Most participants (especially women) in a polygamous reported that the polygamous context is not appropriate for safer sex dialogue since it is difficult to know whether the husband also speaks about safer sex with the other wife/wives. Also women in polygamous relationships are uncertain about the behaviors of their co-wives:

I am not sure whether my husband also talks (about safer sex) to my co-wife. You know I may be discussing (about safer sex) with him (husband) but if he does not talk with the co-wife, it may not help, because I am not sure about the (risk) behavior of the co-wife.

[IDI, Female_ 33 years_Polygamous]

One woman mentioned that she could not imagine that a man would speak to all his wives about safer sex aspects.

P1: how can (safer sex dialogue) be done? Someone with several wives, how can he talk with all of them?

P4: Just like the way she has said, in polygamous [marriage] it is impossible to talk about HIV prevention, because even if you talk with one wife, how about the other wives?

P5: […], moving from one wife to the other is not a joke, you may end up discussing with one wife only but not with all.

[FGD_Females_02]

Safer sex dialogue in polygamous relationships is regarded as appropriate only between the husband and the younger wife since the younger wife is perceived at risk of engaging in extramarital relations.

My husband never talks to me about those issues (HIV and safer sex). We do not talk about those issues! We are adults, he only talks with the younger wife since she is still not mature and can be easily trapped by other men.

[IDI_Woman_43 years_Polygamous]

A male participant reported that he only trusts the older wife (who is like a sister to him). Therefore, he only instructs the younger wife about being faithful in marriage:

I keep an eye to the younger wife and alert her about being faithful. You know the younger wife is young and may be easily deceived to engage in extramarital affairs. I trust the older wife since we have stayed together for a long time. I cannot tell my older wife about those things (HIV prevention) since she is like my sister.

[IDI_Male_40 years_Polygamous]

### Gender power relations

Participants reported that women usually initiate discussions on safer sex by instructing their husbands to abstain from extramarital affairs and being careful with HIV. If this is a shared norm, it could reflect that women despite their engagement in risk sexual behavior take the responsibility of watching over their husband's sexual behavior. However, talking about condom use and couple counseling and testing may lead to conflict and divorce:

When you talk about condom use or going for couple counseling and testing you will bring conflict in the marriage and even divorce. In order to avoid those things (conflict and divorce) you just keep quit. Even in the context where a woman has been tested and found positive, she will never disclose that to the husband, so she will just continue to infect the husband.

[FGD_Females_03]

Women who reported to have been instructing their husbands about abstaining from extramarital affairs stated:

For us we do not discuss (about HIV prevention), but I am the one who usually tells my husband that he needs to be careful with HIV, look we can both die and leave our kids, but he never listens.

[IDI_Female_35 years_Monogamous]

One male participant admitted that it is his wife who usually initiates the safer sex discussion and he cannot initiate the discussion since he is the one engaging in extramarital affairs:

Like in my marriage, my wife is the one who usually initiates the discussion about safer sex. She normally tells me that I should not go outside my marital. It is always hard for me to initiate those discussions because I normally go out with other women.

[IDI_Man_ 26 years, Monogamous]

### Conceptualization of safer sex

Safer sex practices were mainly linked to abstinence from extramarital affairs. Knowledge of condom use and couple counseling and testing was not common. This could be due to marital normative aspects presented above.

P: In my opinion, I think that safer sex is abstaining from other sexual partners outside your marriage.

I: What about condoms?

P: He (husband) does not want to hear about it (condom) and I cannot force him since I will be in trouble (may be beaten or threatened to be forced out of marriage).

[IDI_Female_40 years_Monogamous]

## Discussion

Our findings suggest that open safer sex dialogue between monogamous and polygamous partners in Ifakara town, Tanzania is challenged by interrelated multiple social-cultural factors. Findings are divided into: 1) relationship quality; 2) social norms of marital relation; and 3) gender power relations.

### Relationship quality

In this study, we observed that the participant's view of ‘happy marriage’ had a symbolic meaning as it would not necessarily translate into a resource to safer sex dialogue and practice. This could possibly reflect how spouses in marriages are culturally obliged to use symbolic expressions such as ‘happy marriage’ to protect the positive images of their marriages even in the context of marital uncertainties and risk behaviors. Hopkins and Lewis (page 162) (48) noted that in some cultures a woman is expected to retain a peaceful marriage regardless of marital disputes. Leaving a marriage would bring shame upon the paternal name and is considered being contrary to the spirit of women's tolerance. However, women's tolerance is context specific. A study (49) in Uganda concluded that while urban women would not tolerate conflicts in their marriage, rural women preferred not to leave their marriage regardless of the circumstances.

In our study, men's portrayal of their marriage as happy marriage despite their extramarital behaviors, may have emanated from the expected masculine view in Tanzania, which also considers men's extramarital behavior as a sign of being a ‘complete man (strong)’ (50).

This discussion lays the foundation of how further safer sex dialogue between married partners is constructed from various social-cultural dimensions.

Our study also showed that marital relationship uncertainties (marital conflicts, sexual dissatisfaction, safeguarding trust, and extramarital affairs) challenged an open safer sex dialogue between partners. This finding is similar to dynamics observed in Zimbabwe and Malawi where trust, and a belief that condom use would contradict love, constrained safer sex negotiation and practice for married partners (51, 52). Parker et al. (53) in South Africa found that concerns about partners’ infidelities, that is, extramarital affairs, were a barrier to safer sex dialogue among couples.

Our results highlight the role of relationship quality as a barrier to safer sex dialogue. This is a strong challenge to the notion of economic vulnerability being the main aspect constituting HIV vulnerability among women (54, 55), since women in marriage may experience other aspects of HIV vulnerability beyond economic such as poor relationship quality. Unfortunately, relationship quality is a rare theme in most of the HIV intervention programs. This could be due to its complexity and being too ‘distal’ from commonly accepted HIV risks (9). However, knowledge on the distal determinants of HIV risk is now well recognized to be important for the design of long-term approaches to HIV vulnerability (6–9).

### Social norms

#### Norms of marital relation

We found that norms of marital relations imply that married partners are obliged to comply with social expectations linked to marriage even when the expectations contradict safer sex practices including safer sex dialogue. These findings are credible in light of other studies in SSA. In Zimbabwe, extended family members and religious leaders explicitly or implicitly discouraged women's safer sex negotiation with their husbands (51). Traditional beliefs in Malawi prevented married couples from supporting condom use (56). Social norms in Nigeria were a barrier for married men to participate in prevention of mother-to-child transmission services (PMTCT) including couple counseling and testing services (57). Gagnon and Simon (58) argues that in most societies social norms influence sexual communication through a structured set of behavioral guidelines that create cultural norms for how sex and sexuality can be expressed. Beyond their influence on safer sex dialogue, social norms as reflected by gender expectations significantly influenced HIV status of married and cohabiting men and women in Tanzania (12). Karim et al. (11) also found that it was difficult for married partners to influence condom use because of the dominant ideologies about marriage.

### Norms of marital status

#### Polygamous context

We observed a belief that ‘it is only the younger wife who is considered worthy of safer sex dialogue in polygamous marriage since she is perceived as more vulnerable to risk behaviors based on her age and level of maturity’. Doing so, might risk overlooking the HIV vulnerability of older wives. Women in polygamous relationships also felt that it was useless and difficult to practice safer sex dialogue since they are uncertain about the sexual risk behaviors of co-wives. Only few studies exist in this field. We, however, consider these observations as of critical importance since they reflect the realities of marital context, how they shape practice of safer sex dialogue, and the understanding of who is vulnerable to HIV. Cradock (59) highlight that in the dominant HIV prevention discourse, individuals are framed with a particular identity or position, overlooking the fact that other identities or positions also contribute to their vulnerability. Likewise, the public health discourse may overlook that individuals have their own context-based perspectives of HIV vulnerability. This oversight could partially explain why some couple-based programs have not yet fully succeeded.

Although the SDH framework highlights culture and social values as social-structural determinants of health, the social norms are not clearly defined. Despite their similarity, these concepts are different. Culture reflects people's ways of life (60). Social values denote what people consider being of importance and influence their practices (61). Social norms reflect the rules that dictate what people should do and what they should not do in a particular society (29). Therefore, the social norms have a dictating connotation – a reflection of how powerful they could be in shaping health decisions.

### Gender power relations

Some female participants in our study pointed out that dialogue on couple counseling and testing or condom use may subject them to violence or divorce. This is a reflection of existing gender inequality in marriage and of violation of human rights. The Demographic and Health Survey report in Tanzania indicates that about half (41%) of the surveyed married individuals were likely to be exposed to gender-based violence (62). Elsewhere in SSA, it was found that women are voiceless negotiating for safer sex due to fear of perceived or actual consequences, which include violence and abandonment (51). Other studies show that power in gender relations places men in control of when, where, and how sex takes place (63). In South Africa a study showed that women were more likely to use condoms if they had more gender-equal views, compared with women whose views were male dominated (64).

It emerged from our study that women in monogamous relations may take a lead in instructing their husbands to abstain from extramarital affairs. We would expect the same among women in polygamous relationships. However, as pointed out earlier, a polygamous marriage is perceived as not conducive for safer sex dialogue. Nevertheless, the finding that women in monogamous relationships take the lead in instructing their husbands to abstain from extramarital affairs is contrary to our expectation since in SSA women may not be expected to raise their voices to men regarding sexual matters (65). It seems, however, that in Tanzania, having more women entering the informal sector as entrepreneurs (66) is now challenging this culture. As such, having more women joining the Village Community Bank (VICOBA) in Ifakara town, as informally observed in our study community may have improved women's ability to communicate extramarital behaviors of their husbands. But this assumption requires further investigation.

### Implications

The current call to address HIV vulnerability in a long-term, requires understanding of how social-structural factors influence various patterns of safer sex practices (6–9). This study reflects some of these social-structural aspects that limit open safer sex dialogue between spouses. Based on these findings, it is not surprising that, despite intensive promotion of couple counseling and testing, its uptake is still low in Tanzania (67–69).

Our findings therefore support the argument that besides emphasis on the individual-behavior approaches to safer sex communication and practices, there is a need to address social norms about marriage, relationship quality, marital type, and gender power relations. These marital and community level constructs are likely to challenge not only safer sex dialogue but also the uptake of other proven HIV prevention interventions in marriage like early antiretroviral therapy (ART).

Health promotional messages in Tanzania should advocate for safer sex communication rights of men and women in polygamous and monogamous relations, and reflect on social norms and expectations that constrain partners’ mutual dialogues on condom use and couple counseling and HIV testing services. In addition, gender-based violence should be targeted in parallel with emphasizing the importance of quality relations.

Some of the social-structural challenges to safer sex dialogue are complex and may require creating wide opportunities for women's social and economic development programs, and policies that support men and women's rights to participate in safer sex dialogue regardless of their positions in marriage.

## Conclusion

This study demonstrates that social norms regarding marriage, gender power relations, relationship quality (marital conflict, extramarital affair, sexual dissatisfaction), and marital types (polygamous/monogamous) challenge open safer sex dialogue between spouses living in polygamous and monogamous marriages in Ifakara town, Kilombero district, southeastern Tanzania. Moving beyond the current behavior-centered paradigm by considering contextual factors is key for better understanding of the underlying determinants of safer sex communication and other aspects of HIV risk among married individuals.

The WHO-SDH framework is a useful approach for understanding how various social-structural determinants influence health and health inequities. In the context of safer sex dialogue, the framework should explicitly reflect relationship quality, marital status, social norms, and gender power in stable relations.

## Paper context

Behavior change approaches to address HIV vulnerability among married spouses in Tanzania encourage partners to speak about safer sex. Drawing on the WHO Social Determinants of Health framework, this study explored key social-structural constructs that influence safer sex dialogue in polygamous and monogamous relations in southeastern Tanzania. Findings highlight that marital relationship quality, social norms about marriage, marital status (polygamous), and gender power relations determine safer sex dialogue and need to be addressed in HIV prevention interventions.
